# ‘Acridines’ as New Horizons in Antifungal Treatment

**DOI:** 10.3390/molecules25071480

**Published:** 2020-03-25

**Authors:** Iwona Gabriel

**Affiliations:** Department of Pharmaceutical Technology and Biochemistry, Gdańsk University of Technology, 80-233 Gdańsk, Poland; iwogabri@pg.edu.pl; Tel.: +48-58-3486-078; Fax: +48-58-3471-144

**Keywords:** antifungals, topoisomerase, inhibitor, acridine, acridone, biofilm, photoantimicrobials, morphological transformation

## Abstract

Frequent fungal infections in immunocompromised patients and mortality due to invasive mycosis are important clinical problems. Opportunistic pathogenic *Candida* species remain one of the leading causes of systemic mycosis worldwide. The repertoire of antifungal chemotherapeutic agents is very limited. Although new antifungal drugs such as lanosterol 14α-demethylase and β-glucan synthase inhibitors have been introduced into clinical practice, the development of multidrug resistance has become increasingly significant. The urgency to expand the range of therapeutic options for the treatment of fungal infections has led researchers in recent decades to seek alternative antifungal targets to the conventional ones currently used. Among them, many compounds containing an acridine scaffold have been synthesized and tested. In this review, the applicability of acridines and their functional analogues acridones as antifungal agents is described. Acridine derivatives usage in photoantifungal chemotherapy, interactions with fungal transporters resulting in modulation of efflux/influx pumps and the effect of acridine derivatives on fungal topoisomerases are discussed. This article explores new perspectives on the mechanisms of antifungal acridine-peptide conjugates and acridine-based hybrid molecules to effectively combat fungal infections.

## 1. Introduction

**Figure 1:**
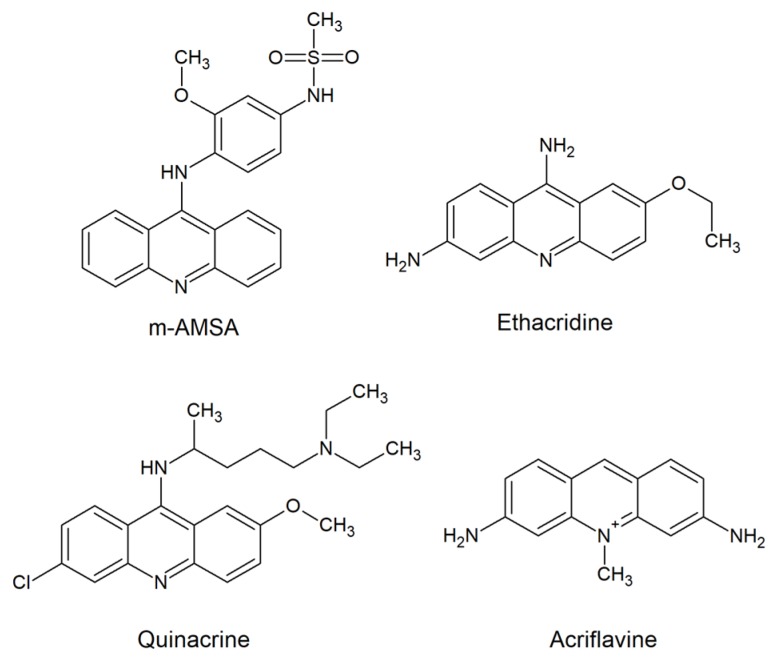
Biologically active molecules based on acridine nucleus used in clinic [1,9].

The first clinical use of acridines as antimicrobial agents occurred in 1917. Among compounds containing the acridine chromophore, aminoacridines were found wide use, both antibacterial (e.g., ethacridine; Figure 1) and as antimalarial (e.g., quinacrine; Figure 1) agents [1].

Due to the great therapeutic efficacy of penicillins, the use of acridines for antibacterial therapy was discontinued. Thus, acridine-based drugs have remained as antiseptics. However, compared to antibiotics for bacterial infections, advances in the treatment of fungal infections have been slower. In contrast to bacteria, fungal cells are similar to mammalian cells because of their eukaryotic origin. The problems in treating fungal infections are in many ways similar to those faced in developing treatments for cancer [2]. Therefore, it is highly challenging to identify targets that would be fungal-specific and develop selective drugs. The first strategy for novel antifungal drug discovery and development is to optimize and improve existing drugs. The second is to identify new targets specific for fungal cell and design effective inhibitors. As far as new targets are concerned, enzymes important for the biosynthesis of fungal proteins, essential amino acids or DNA are widely analyzed [3,4,5,6,7]. Moreover, the development of resistance, often multidrug resistance (MDR), is an emerging problem. Due to widespread use of common antifungals in the treatment of fungal infections, the percentage of resistant yeast strains has grown in the last years [8]. The lack of effective and, at the same time, selective antifungal drugs, the development of fungal resistance and the spreading of fungal infections with high mortality rates, are the obvious driving forces stimulating the search for new treatment strategies.

The ability of acridines to intercalate into DNA has prompted their use as molecular targets for antibacterial and antimalarial aminoacridines [1]. The development of acridine-based derivatives for modern anticancer therapy was also based on the belief that this mechanism of action determines the effect, e.g., amsacrine (m-AMSA, Figure 1) [9]. However, the actual site of action of such a compound is now established at the level of human topoisomerase inhibition, rather than DNA itself. Antitumor activity is acquired by the stabilization of the enzyme-DNA cleavage complex. Surprisingly, although acridine and acridone derivatives are widely analyzed as antimicrobials or anticancer agents, only a few reports have demonstrated their antifungal activity. Thus, this review briefly discusses the applicability of such compounds as antifungals, their multifunctional mode of action and new perspectives for acridine-based hybrid molecules to effectively combat fungal infections.

## 2. Acridine Derivatives as Antifungals

### 2.1. Antifungal and Antibiofilm Activity

Acriflavine (Figure 1) is an antiseptic and effective agent against parasitic infections [1]. It is also used as a fungicide. As reported previously, it was able to induce mutations in the yeast *Saccharomyces cerevisiae* [10] and kinetoplast loss in Trypanosomatidae [11]. Results obtained by Keyhani et al. showed that in the yeast *Candida utilis*, acriflavine (at 30–180 μM concentration) caused apoptosis and necrosis [12]. Its presence revealed an alteration in fungal cell respiratory control ratio and a decrease in cytochrome content. The microscopic evaluation of cells treated with acriflavine showed that apoptotic cells exhibited chromatin condensation and cytoplasmic lysis. In contrast, necrotic cells showed no distinctive intracellular organelles. The results indicated that apoptotic death of fungal cells was mediated by a change in mitochondrial permeability and cytochrome c release or by plasma membrane death receptor activation. It was shown that an efflux of protons to the cytosol and cytoplasmic acidification caused an electrochemical gradient disturbance, a decrease in adenosine triphosphate (ATP) synthesis and cytolysis.

**Figure 2:**
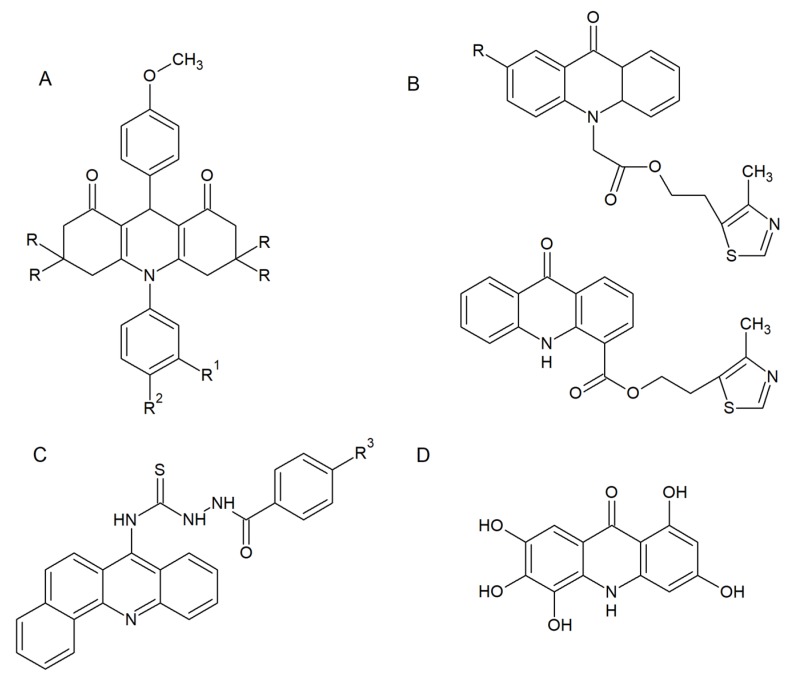
Selected derivatives with antifungal activity: (**A**) 1,8-dioxoacridine derivatives; (**B**) 2-(4-methyl-1,3-thiazol-5-yl) ethyl esters of acridone carboxylic acids; (**C**) acridine thiosemicarbazide derivatives; (**D**) M14 compound. R: –H or –CH_3_; R^1^: –F, –Cl, –Br, –J or –CH_3_; R^2^: –H or –COOH; R^3^: –H, –Cl, –NO_2_, –OCH_3._ [13,14,15,16].

In view of the fact that such derivatives are attractive frameworks for further development as antimicrobial agents, many scientists seek for effective, more selective and less toxic antifungal drugs among acridine derivatives. Kaya et al. presented a synthesis and biological evaluation of acridine derivatives as potential antimicrobial agents (Figure 2A) [13].

Most of the compounds showed significant activity against “Gram-positive” and “Gram-negative” bacterial strains (*Escherichia coli*, *Pseudomonas aeruginosa*, *Salmonella enteritidis*, *Staphylococcus aureus*). Additionally, all derivatives exhibited moderate antifungal activity (*Candida glabrata* and *Candida albicans*) [13]. Markovich et al. described the synthesis of 2-(4-methyl-1,3-thiazol-5-yl) ethyl esters of acridone carboxylic acids (Figure 2B). These compounds were tested for antimicrobial activity against bacterial (*P. aeruginosa*, *E. coli*, *Proteus vulgaris*, *S. aureus*, and *Bacillus subtilis*) and fungal (*C. albicans*) strains. The prepared analogues were active and showed inhibition against all evaluated microorganisms [14]. Acridine thiosemicarbazide derivatives (Figure 2C) exhibited both antibacterial and antifungal activity (minimal inhibitory concentration (MIC) range 10–80 μM) as well as anticancer activity (IC50 range 10–70 μM) [15]. Although the results obtained confirmed the antifungal activity of all of the above-mentioned analogues, the probable mode of action is associated with DNA intercalation ability with the consequences for microbial cell development without being specific for fungal cells.

Recent studies have allowed researchers to obtain a selective derivative M14 (Figure 2D) with anticandidal and anti-dermatophyte activity and low toxicity to human fibroblasts [16]. M14 exhibited growth-inhibitory activity against all reference and clinical strains of *Candida* and dermatophytes (MIC range 7.81–31.25 μg mL^–1^). Moreover, M14 activity was fungicidal; the presence of the acridine derivative prevented *C. albicans* biofilm formation and reduced the viability of preformed biofilm at concentrations lower than MIC. Microscopic evaluation of *C. albicans* hyphal growth in the presence of M14 revealed that the yeast-to-mycelia transformation was completely inhibited. Similarly, severe inhibition of hyphal growth of *Trichophyton rubrum* was shown [16].

Quinacrine (QNC; Figure 1), a highly active therapeutic agent still used against giardiasis, was also reported to exhibit antifungal activity. Recent studies have indicated that QNC showed the highest activity against planktonic *Candida* cells at alkaline pH. High doses of that acridine derivative prevented *C. albicans* biofilm formation by several independent mechanisms. First of all, QNC dramatically inhibited the yeast-to-mycelia transformation. Moreover, antibiofilm activity also occurred by vacuolar alkalinization and defects in endocytosis [17].

The ability of fungal cells to perform morphological transformation is one of the most important virulence factors. As revealed by RNA-sequencing analysis of *T. rubrum* transcriptome in response to sublethal doses of acriflavine (Figure 1), 490 unique genes were modulated. Among them, the modulation of 69 genes was observed. As indicated by functional categorization of these genes, their putative role in various cellular processes was shown. The presence of acriflavine affected transmembrane transport, oxidation-reduction reactions and metal ion binding. Interestingly, genes putatively involved in the pathogenicity of dermatophytosis were downregulated. Results indicated that this drug targets virulence factors of *T. rubrum*, which are necessary for the establishment and maintenance of fungal infection in the host [18].

*Candida* spp. yeast-to-mycelium transformation and its ability to form a heterogeneous biofilm structure are associated with the ability to cause infection [19]. In recent years, awareness of the role played by fungal biofilms in human diseases has increased. Unique phenotypic characteristics of microbes growing within biofilms compared to their planktonic counterpart cells have been observed. The most important with respect to antifungal agents seems to be its increased resistance to antimicrobial agents [20]. As traditional antifungals act by inhibiting the growth of fungal cells or killing them, targeting virulence factors represents a promising alternative. This approach may potentially influence selective pressure by reducing, eliminating or even reversing it, and thus overcome drug resistance [21]. In light of these data, acridine derivatives that block morphological transformation or biofilm development constitute an interesting group of antifungal compounds.

### 2.2. Acridine/Acridone Derivatives as Efficient Sensitizers

Photoantimicrobials, also known as photosensitizers, are commonly known antimicrobial agents that can be activated by light in a process called antimicrobial photodynamic inactivation (PDI) [22]. Their mode of action is related to the ability to produce highly reactive oxygen species (ROS) in the presence of oxygen. ROS are very toxic to microbial cells and can act on multiple targets such as nucleic acids, proteins or unsaturated lipids. Although it seems to be a disadvantage due to their short lifespan, ROS are active locally. The multiple-target action of these compounds is therefore beneficial because photoantimicrobials are relatively immune to individual resistance mechanisms. As a consequence, these agents can act effectively against wild-type and resistant strains [23,24]. Moreover, the selectivity of photoantimicrobials for fungi over human cells has been demonstrated in recent years; so far, no reports of fungal resistance exist. No genotoxic or mutagenic effects on fungi or human cells have been reported [25].

**Figure 3:**
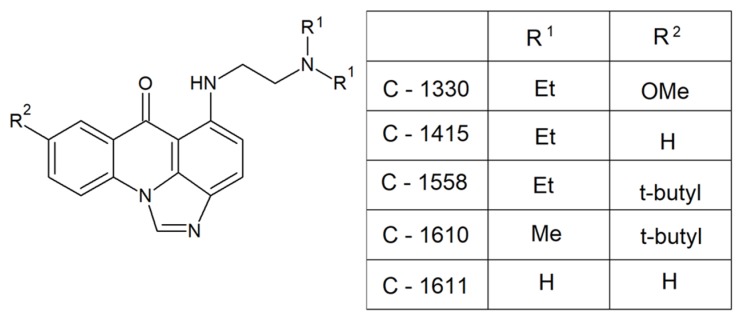
Efficient photosensitizers in photodynamic inactivation (PDI) of *C. albicans* [27]. Et, –CH_2_CH_3_; Me, –CH_3_; OMe, –OCH_3_; t-butyl, –C(CH_3_)_3._

Acridine and acridone derivatives are molecules with fluorescence properties that have also been demonstrated to possess photosensitizing properties [26,27,28]. Studies performed by Taraszkiewicz et al. showed that imidazoacridinones (Figure 3) were efficient photosensitizers in the PDI of *C. albicans* and significantly inhibited the growth of planktonic cells [27].

The highest photokilling efficacy was observed for C-1330, which correlated with an efficient accumulation in the nucleus and vacuoles. Microscopic evaluation of *C. albicans* cells incubated with that compound upon illumination revealed significant changes in above mentioned intracellular structures. Importantly, approximately 40% of analyzed human cells survived the treatment, demonstrating selectivity of C-1330 in photoantimicrobial chemotherapy.

**Figure 4:**
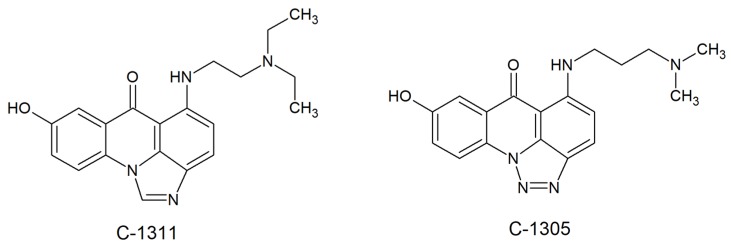
Human topoisomerase II inhibitors: triazoloacridinone (C-1305) and imidazoacridinone (C-1311) [29].

Acridines/acridones and their derivatives are considered to be antimicrobial agents, although their activity is obviously determined by their efficient accumulation in microbial cells. As described for imidazoacridinone C-1311 (Figure 4) and its nine derivatives, only three of those that entered in fungal cells (C-1330, C-1415 and C-1558) showed phototoxic antifungal activity (Figure 3).

Despite the high efficiency of C-1311 as an anticancer compound that intercalates into DNA and inhibits human topoisomerase II [29] (was in phase II clinical trials for the treatment of breast cancer [30]), it was unable to accumulate in *C. albicans* cells and no antifungal activity was observed [27]. Results of experimental investigations demonstrated that the fungicidal activity, which is light-induced, might depend on the production of superoxide anions [28]. The highest rate of superoxide anion generation was observed for C-1330 (hydrochloride salt), C-1610 (hydrochloride salt) and C-1611 (Figure 3). Moreover, irradiation of imidazoacridinone derivatives with UV laser light indicated the presence of singlet oxygen with a low to moderate yield, depending on the type of compound. Thus, compounds analyzed by Taraszkiewicz et al. might have acted by using a mixed type of photodynamic mechanism. Importantly, no direct correlation between the structures of studied imidazoacridinone derivatives, cell killing ability and ROS production was observed. For the most effective compounds capable of killing fungal cells by PDI, not only intracellular accumulation was necessary, but also localization within particular cellular structures [28].

Summing up, the results clearly indicate that fungal cells can be effectively killed by photodynamic inactivation using acridine-based photosensitizers. Importantly, the selectivity of photoantimicrobials for fungi over human cells has been demonstrated. In light of these data, photodynamic therapy with the use of acridine/acridone derivatives reveal potential to be developed as therapy for difficult-to-treat fungal infections of accessible regions of the body. It might be possible to threat deep fungal infections using fiber optic lasers, applied endoscopically or interstitially [31].

### 2.3. Fungal Topoisomerases as Drug Targets for Acridine/Acridone Derivatives

Topoisomerases are enzymes that can control the topological state of DNA during transcription, replication and chromatin condensation [32]. The mode of action involves introducing transient enzyme-bridged DNA breaks that allow for passage of DNA strands. There are two classes of topoisomerases, which differ in terms of single strand (type I) or double strand (type II) DNA breakage.

Acridine and acridone derivatives are widely analyzed as human topoisomerase inhibitors for cancer chemotherapy. Amsacrine (m-AMSA) (Figure 1), obtained by Denny’s group [33,34], was the first synthetic drug approved for clinical usage that was shown to act as a topoisomerase inhibitor. There has been substantial work showing that the mode of action of m-AMSA as DNA intercalation ability was not the only prerequisite for the activity. Structural analysis of topoisomerase II complexes revealed strong interaction of amsacrine with topoII-DNA [35] and allowed researchers to obtain m-AMSA derivatives with stronger antitumor activity and weaker side effects.

Although its mechanism of action is still being investigated, an acridine derivative, triazoloacridinone (C-1305, Figure 4), was shown to demonstrate strong inhibiting properties toward human topoisomerase II in vitro [36,37]. Similar to triazoloacridinone, antitumor imidazoacridinone C-1311 (Figure 4) was able to inhibit the cell cycle in the G2 phase. The molecular mechanism indicated its intercalation with DNA as well as the formula of a topo II-stabilizing complex [38,39]. The success of anticancer and antibacterial drugs as DNA topoisomerases inhibitors [40] highlights the potential of topoisomerases from fungal cells as targets for the development of novel antifungals.

Significant work has been done on the structure and function of topoisomerase I and II in fungi. Results indicated that their activity is crucial for some strains and it depends on the species. The two topoisomerases from *S. cerevisiae* serve separate functions which are important to cell transcription, replication, and recombination [41,42], although topo II was reported as essential for viability [43], whereas topo I was not [41]. Studies performed with *C. albicans* and *Cryptococcus neoformans* suggested that for both fungal pathogens topoisomerase I was essential. Importantly, the fungal and mammalian topoisomerase I enzymes exhibit structural differences; their primary structures contain amino acid insertions not found in the mammalian enzyme. The functions of the fungal inserts are not yet known but the selectivity of fungus-specific topoisomerase I inhibitors seems probable [44,45].

The effect of known human topoisomerase inhibitors on the growth of fungal cells was analyzed by Kwok et al. and Steverding et al. [46,47]. Within the anticancer drugs tested, only two displayed antifungal activity. Aclarubicin and idarubicin were reported to be effective against *Aspergillus niger*, *C. glabrata*, *C. albicans* and *C. neoformans*, with MICs varying between 1.8 and 8.4 μg mL^–1^. Results obtained by viability assay showed that aclarubicin mode of action was fungistatic. Microscopic evaluation of *C. albicans* hyphal growth in the presence of four topoisomerase inhibitors (daunorubicin, doxorubicin, idarubicin and β-lapachone) revealed that the yeast-to-mycelia transformation was strongly affected.

m-AMSA (Figure 1), etoposide and its derivatives A-80198 and A-75272 (a tricyclic quinolone) were analyzed by using isolated DNA topoisomerase II from *C. albicans* [48]. The results were compared with those obtained for mammalian enzyme. The inhibitory effect observed in the presence of m-AMSA, etoposide and the derivative A-80198 was greater for calf thymus topoisomerase II than its fungal counterpart. On the other hand, A-75272 showed the opposite outcome. The fact that fungal and mammalian enzymes responded in a different manner to the studied compounds suggests that there are sufficient biochemical differences to obtain selectivity for fungi over human cells. Selective targeting was also suggested by Fostel et al. for topoisomerase I [49]. Kwok et al. demonstrated no antifungal activity of etoposide [46], although its inhibitory effect on *C. albicans* DNA topoisomerase II was previously reported [48]. Thus, in order to be active, antifungal topoisomerase inhibitor probably needs to enter into fungal cells to reach their intracellular targets.

Even though acridine and acridone derivatives are widely analyzed as human topoisomerase inhibitors for cancer chemotherapy, only a few reports have demonstrated its antifungal activity. Fungal topoisomerases are sufficiently distinct from their human counterparts to enable selective targeting and are therefore a good target for antifungal drug discovery. Obviously, clinical application of acridine-based compounds is limited because of the side effects. However, with the current development of multidrug resistance, new derivatives are highly desired.

### 2.4. Antifungal Acridine-Peptide Conjugates

Peptide–acridine conjugates (PACs) constitute an extremely important family of compounds in antitumor chemotherapy [50]. Conjugates of acridines or acridones in antimicrobial therapy have not been so widely analyzed, although their highly valuable mode of action was discovered. A significant improvement over DNA intercalating acridine binders was demonstrated for peptide derivatives with amino acid sequences localized at positions 4 and 9 of the tricyclic ring [51,52]. PACs were recognized to selectively bind RNA, according to the mechanism named “threading intercalation.” This type of binding is specific and targets RNAs, especially RNA aptamers that possess a CpG sequence and still not fully defined duplex secondary structures. It was proved that PACs target RNAs by stabilizing interactions between substituents on the intercalator and functionality present in the grooves of the duplex or at bulged, mismatched or looped nucleotides found adjacent to the intercalation site [51].

Combining acridines and short cationic peptides with antimicrobial properties (antimicrobial peptides (AMPs)) led to the discovery of compounds that exhibited a dual mechanism of action. The ability of these compounds to kill microbial cells by membrane disruption, based on pore formation on one side and intracellular DNA binding as the second target, gave them individual characteristics and greater efficacy in antimicrobial therapy. A highly active antimicrobial peptide was synthesized by Zhang et al. [53]. Conjugates of acridines with an N-terminus of a nuclear localization sequence (NLS, PKKKRKV) displayed high antibacterial activity and strong bactericidal action. Importantly, the selectivity of the analyzed compounds for microbial over human cells was demonstrated though they presented low toxicity at much higher concentration than their MIC values [53]. In light of these data, it is possible to use higher concentrations to overcome infections, which is very important for the clinical development of antimicrobial agents.

**Figure 5:**
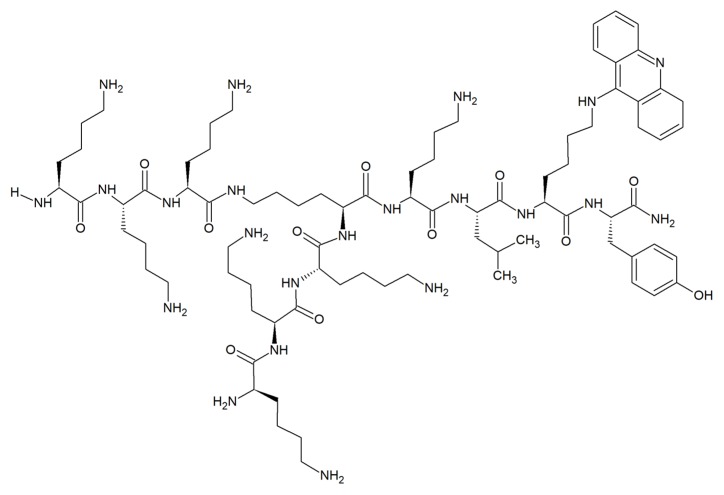
Example of acridine-peptide conjugate with strong antifungal and antibiofilm activity and no hemolytic effects [54].

Surprisingly, very few examples of acridine-peptide conjugates exhibiting antifungal activity have been described. Recent studies indicated that branched peptide acridine derivatives are a promising group of antimicrobial compounds with antifungal activity (Figure 5) [54].

Hydrophobic acridine-based conjugates with cationic lysine moieties were reported to display amphipathic characteristics commonly seen with AMPs. Compounds were active against *S. aureus*, *C. albicans* and *E. coli* with MICs as low as 1 μg mL^–1^ and significantly inhibited growth and biofilm formation of *C. albicans*. The branched peptide acridine derivatives were nonhemolytic and nontoxic to mammalian cells even at concentrations 10 times higher than MICs. Interestingly, no effect on fungal membrane integrity at MIC of acridine-conjugated derivatives and their corresponding control sequences was observed, and removal of acridine moiety did not affect the strong antifungal activity against *C. albicans*. In contrast, the moderate inhibition against *A. niger* was related to the presence of acridine scaffold, as its removal led to the loss of activity. Thus, the mechanism of action of branched peptide acridine derivatives against fungal cells did not mainly involve intercalation of acridines into nucleic acids or membrane disruption [54].

The clinical treatment of fungal infection is associated with overcoming several challenges, such as MDR and a limited number of fungal-specific cellular targets. The use of peptide acridine conjugates seems to be promising in light of the results obtained for multidrug-resistant strains. Clinical isolates of *C. albicans* and genetically modified *S. cerevisiae* cells that overexpress genes encoding *C. albicans* drug efflux pumps were reported to be more susceptible to several different antifungal oligopeptides. The enhanced susceptibility of yeast strains overexpressing ATP-binding cassette (ABC) multidrug efflux pumps to oligopeptide antifungals corresponded to the higher rates of oligopeptide uptake [55]. On the other hand, recent studies performed with verapamil, a known inhibitor targeting *Candida* ABC transporters, suggested that imidazoacridinones are not substrates for ABC efflux pumps. In contrast to azoles, a major class of antifungal agents used to treat candidiasis, ABC transporter-mediated resistance is unlikely [28].

### 2.5. Acridine Interactions with Efflux/Influx Pumps

**Figure 6:**
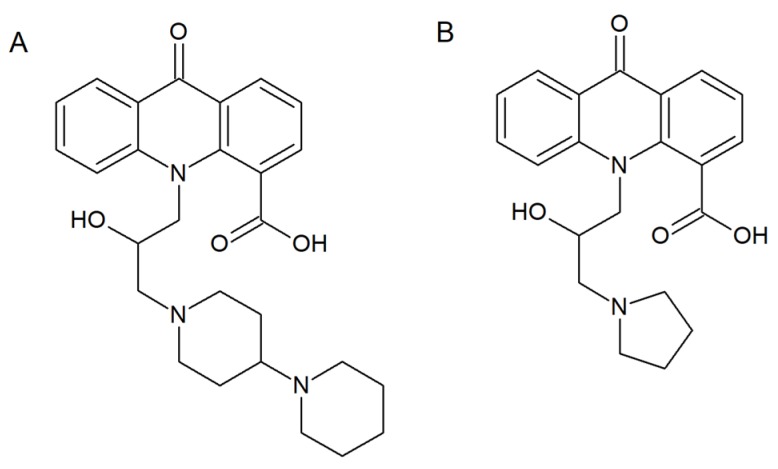
Compounds (**A**,**B**) with multidrug resistance modulating properties [57,58].

Overexpression of ATP-binding cassette (ABC) transporters or membrane proteins belonging to the major facilitator superfamily (MFS) is the molecular mechanism underlying multidrug resistance. As far as *C. albicans* cells are concerned, two protein transporters, Cdr1p and Cdr2p, are the major ABC drug efflux pumps, while the main representatives of MFS are *Ca*Mdr1p and FLU1p [56]. Singh et al. synthetized several acridone derivatives and analyzed their antifungal activity and influence on Rhodamine6G (R6G) influx/efflux in the *C. albicans* CAI4 strain [57]. Molecular docking investigations revealed that the introduction of a –COOH or –Cl substituent at the C-4 position of the acridone scaffold enhanced their interactions with components of membrane transporters. The most significant results were obtained for acridone analogue (A) (Figure 6). Fungal cell growth inhibition by a cell rupturing mechanism was also associated with increased influx and efflux of R6G in the presence of (A).

Subsequent in vitro and in vivo analysis supported the applicability of another compound (B) (Figure 6), as the most suitable MDR modulator. Results indicated that the presence of compound (B) decreased the efflux of R6G from the *Candida* cells. It might occur through the inhibition of the two major *Candida* protein transporters, Cdr1p and Cdr2p. As suggested by the authors, compound (B) is a suitable candidate for further study for multidrug resistance modulating properties [58].

As reported previously, RNA-sequencing analysis of *T. rubrum* transcriptome in response to the presence of acriflavine (Figure 1) revealed differentially expressed genes involved in transmembrane transport [18]. Most of these genes were downregulated, whereas *MDR4* was upregulated. A higher affinity of Mdr4p to the drug was therefore suggested. It is also possible that this protein transporter was involved in the efflux of acriflavine from the cell in order to enhance cell viability. In contrast to *MDR4*, another gene, *TruMDR2*, was downregulated. The Mdr2p transporter, belonging to the multidrug transporters of the ABC family, is involved in *T. rubrum* resistance to some antifungal drugs. The decreased expression of this gene and others during acriflavine treatment suggests that the drug interferes with key processes that allow cell detoxification and growth [21].

## 3. Conclusion and Future Perspective

The frequency of fungal infections and mortality due to invasive mycosis remains an important clinical problem. New drug targets and novel antifungal agents are therefore urgently needed. To date, thousands of acridines with therapeutic and biological activity have been developed. A feeling of mistrust in the use of acridine derivatives is related to their well-known high toxicity associated with DNA intercalation. This site of action is well known for simple aminoacridines as antibacterials. However, in many cases compounds based on the acridine chromophore are non-intercalators. In addition, intercalation into microbial DNA is not necessarily the same in human cells. There are many new chromophores based on the acridine scaffold with modified intercalative ability. Derivatives with antifungal activity and low cytotoxicity to human cell are therefore reported (Table 1).

Unlike bacteria, eukaryotic fungal cells are similar to mammalian cells. A number of targets involved in the cell membrane and protein (or DNA) biosynthesis are not fungal-specific. Therefore, developing selective drugs is highly challenging. Selectivity can be achieved by fungal topoisomerase targeting. Those enzymes are probably sufficiently distinct from their human counterparts, making them a good target for antifungal drug discovery. Surprisingly, although acridine and acridone derivatives are widely analyzed as human topoisomerase inhibitors for cancer chemotherapy, only a few reports have demonstrated their antifungal activity. Thus, further validation of these novel targets by designing, synthesizing and evaluating the activity of new inhibitors is highly valuable.

In contrast to compounds that act on traditional targets by inhibiting the growth of fungal cells or killing them, targeting virulence factors may be a promising alternative. The ability to form a heterogeneous *Candida* biofilm structure is one of its major virulence attributes. These biofilms exhibit unique phenotypic characteristics compared to planktonic cells, particularly increased resistance to antimicrobial agents. Acridine derivatives that block morphological transformation or biofilm development constitute an interesting group of antifungal compounds (Table 1).

The development of a bacterial or fungal infection is often a serious complication in patients undergoing chemotherapy. Immune deficiency caused by disease or granulocytopenia appearing as a result of aggressive chemotherapy is the main cause of infection. Parental acridines and acridones are potent antitumor compounds. Thus, it is plausible to expect that a new group of derivatives may also display antitumor properties. If the proposed drugs display dual antimicrobial and antitumor activity, this may lead, in a larger perspective, to potential application of new derivatives in cancer patients, helping them to simultaneously fight both cancer cells and microbial infections. The rational design and development of peptide–acridine conjugates is believed to be highly promising and allow for a multitargeted approach. Importantly, it is probable to obtain compounds with synergistic or even additive properties coming from two different compounds combined as one.

**Figure 7:**
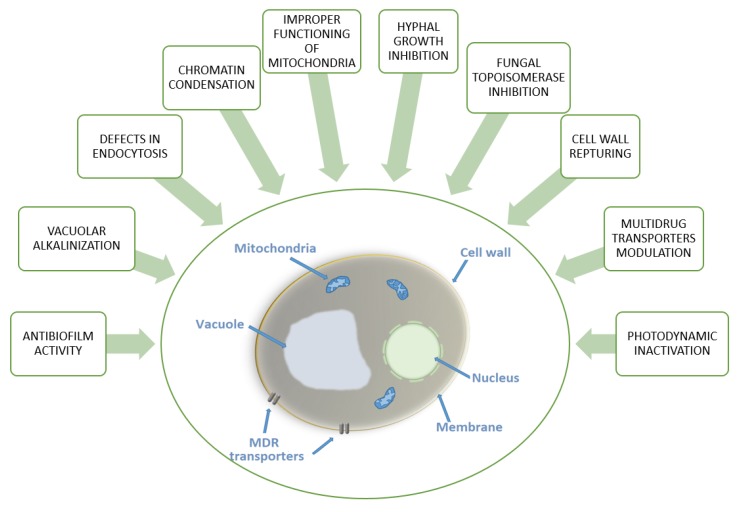
Diagram summarizing reported antifungal modes of action of acridine derivatives.

Summing up, the search for antifungal drug candidates among acridine derivatives is undoubtedly worth continuing. With better understanding of antifungal mechanisms—which may include the processes shown in Figure 7 and increased medicinal chemistry efforts, a new group of acridine-based antifungals may become a reality in the near future.

## Figures and Tables

**Table 1:** Summary of reported antifungals among acridine derivatives and their properties. MIC, minimal inhibitory concentrations.

Compound				
	Antifungal Spectrum of Activity	Mode of Action	MIC range	Ref.
**Acriflavine**	*S. cerevisiae*	Differences in mitochondrial ultrastructure. Considerable variation in mitochondrial enzyme activities.	-	[10]
	*C. utilis*	Alteration in cell respiratory control ratio and a decrease in cytochrome content. Chromatin condensation and cytoplasmic lysis.	-	[12]
	*T. rubrum*	Alteration in transmembrane transport and oxidation-reduction reactions. Down-regulation of genes putatively involved in the pathogenicity.	-	[18]
**Quinacrine**	*C. albicans*	Growth and filamentation inhibition. Antibiofilm activity. Vacuolar alkalinization and defects in endocytosis.	-	[17]
**1,8-dioxoacridine derivatives**	*C. albicans* *C. glabrata*	Moderate growth inhibition.	-	[13]
**2-(4-methyl-1,3-thiazol-5-yl)ethyl esters of acridone carboxylic acids**	*C. albicans*	Moderate growth inhibition.	-	[14]
**Acridine thiosemicarbazides derivatives**	*C. albicans*	Strong growth inhibition.	20–80 μM	[15]
**M14**	*Candida* spp. *C. parapsilosis* *C. orthopsilosis* *C. metapsilosis* *C. albicans* *C. glabrata* *C. neorugosa* *C. tropicalis* *C. lusitaniae* *C. guilliermondii* *Trichophyton* spp. *T. rubrum* *T. mentagrophytes* *T. tonsurans* *T. verrucosum* *T. schoenleinii*	Fungicidal activity. The inhibition of *C. albicans* biofilm formation. *C. albicans* and *T. rubrum* hyphal growth inhibition. Low toxicity to human fibroblasts.	7.81–31.25 μg mL^-1^	[16]
**Imidazoacridinone derivatives**	*C. albicans* *C. glabrata*	Efficient accumulation in the nucleus and vacuoles. Superoxide anion generation. Not effluxed by *Candida* ABC transporters.	-	[27,28]
**Acridine conjugates with branched lysine peptides**	*C. albicans* *A. niger*	Growth and biofilm formation inhibition. Anticandidal activity not affected by the removal of acridine moiety. No effect on fungal membrane integrity. Moderate growth inhibition related to the presence of acridine moiety for *A. niger*. Negligible hemolytic effects on red blood cells. No toxicity to mammalian cells.	1–4 μg mL^-1^	[54]
**Acridones carrying hydroxyl amine substituent at N-10 and COOH at C-4**	*C. albicans*	Growth inhibition. The cell wall rupturing. Multi drug resistance modulators.	132–405 μg mL^-1^	[57,58]
